# Interferons and Their Receptors in Birds: A Comparison of Gene Structure, Phylogenetic Analysis, and Cross Modulation

**DOI:** 10.3390/ijms151121045

**Published:** 2014-11-14

**Authors:** Hao Zhou, Shun Chen, Mingshu Wang, Anchun Cheng

**Affiliations:** 1 Institute of Preventive Veterinary Medicine, Sichuan Agricultural University, Chengdu 611130, China; E-Mails: zhouhao19911030@163.com (H.Z.); mshwang@163.com (M.W.); 2 Avian Disease Research Center, College of Veterinary Medicine of Sichuan Agricultural University, 46 Xinkang Road, Ya’an 625014, China; 3 Key Laboratory of Animal Disease and Human Health of Sichuan Province, Sichuan Agricultural University, Chengdu 611130, China

**Keywords:** interferon, interferon receptor, predicted gene structure, phylogenetic analysis, interplay

## Abstract

Interferon may be thought of as a key, with the interferon receptor as the signal lock: Crosstalk between them maintains their balance during viral infection. In this review, the protein structure of avian interferon and the interferon receptor are discussed, indicating remarkable similarity between different species. However, the structures of the interferon receptors are more sophisticated than those of the interferons, suggesting that the interferon receptor is a more complicated signal lock system and has considerable diversity in subtypes or structures. Preliminary evolutionary analysis showed that the subunits of the interferon receptor formed a distinct clade, and the orthologs may be derived from the same ancestor. Furthermore, the development of interferons and interferon receptors in birds may be related to an animal’s age and the maintenance of a balanced state. In addition, the equilibrium between interferon and its receptor during pathological and physiological states revealed that the virus and the host influence this equilibrium. Birds could represent an important model for studies on interferon’s antiviral activities and may provide the basis for new antiviral strategies.

## 1. Introduction

The interferons (IFNs) comprise type I IFNs (IFNα, IFNβ* etc.*), type II IFN (IFNγ), and type III IFN (IFNλ), based on their genetic, structural, functional features and their cognate receptors [1]. IFNλ is further divided into IFN-λ1 (IL-29), IFN-λ2 (IL-28A) and IFN-λ3 (IL-28B) [2,3]. Interferon receptors consist of type I IFN receptor (IFNAR1 and IFNAR2), type II IFN receptor (IFNGR1 and IFNGR2), and type III IFN receptor (IFNLR1 and IL10R2) [4]. IL-10R, IL-22R, and IL-26R complexes also contains an IL10R2 subunit [5]. Interferons, as a large family of cytokines [6], not only protect cells from viral infection, but also may contribute to the promotion of novel vaccines and adjuvants [7]. To activate the antiviral response, IFNs can trigger the induction of the expression of hundreds of IFN-inducible genes via the janus kinase (JAK) and signal transduction and activators of transcription (STAT) signaling pathway [8]. Typically, type I and type III IFN exhibit an antiviral response in some subtypes of cells [9,10], while type II IFN is primarily involved in immunity, host defense, inflammation and autoimmunity [11]. All interferons are recognized as vital regulatory mediators of the immune response.

During the process of acute and chronic infection, interferons are pivotal in balancing antiviral actions against immunosuppressive effects [12]. Surprisingly, the positive feedback regulation of type I IFN through the interferon receptor boosts the immune system, which is important for a delayed antiviral response, which includes inducing type I IFN and interferon-stimulated genes [13,14]. Indeed, over recent decades, accumulating evidence has suggested that interferons play a pivotal role in antivirus, antitumor, and antimicrobial activities, together with the interferon receptors. Just like a seesaw, the regulatory mechanisms maintain the balance between interferons and interferon receptors; both sides could regulate the level of the other cytokine, thus keeping the host in a stabilized and healthy state. 

In recent years, research into interferons and interferon receptors has mainly focused on mammals. Different types of IFNs have been identified [15,16,17,18] (Table 1), for example, in the Asian elephant [19], giant panda [20], ferret [21] and cattle [22]. However, knowledge of interferons in birds has lagged behind, and the exact number of members of interferon subtypes in birds has not been identified.

**Table 1:** List of the interferons identified in some mammals.

Taxonomy	Gene Name	Species	Accession Number	Reference
Type I IFN	IFNα	Mouse	X01969	[15]
		Giant panda	DQ392967	[20]
	IFNβ	Ferret	KJ831215	[18]
	IFNε	Canine	KC527684	[23]
Type II IFN	IFNγ	Mouse	K00083	[16]
		Asian elephant	EF203241	[19]
		Ferret	Y11647	[21]
		Porcine	X53085	[17]
Type III IFN	IFNλ	Bovine	XM002695050	[22]

Furthermore, various kinds of interferon receptors have been cloned and analyzed in mammals [3,24,25] (Table 2), such as feline [26], ovine [27], bovine [27] and woodchuck [28]. However, as far as we know, there is no information available on the cloning and characterization of interferon receptors in birds, except in chickens. Thus, the identification and characterization of avian interferon receptors is a new area of research, which may pave the way for deciphering the mechanism of interferon-regulated transcription in birds. Up to now, little information is available about the mutually regulatory feedback loop between interferons and their receptors in birds.

**Table 2:** List of the interferon receptors identified in some mammals.

Taxonomy	Gene name	Species	Ligand	Accession Number	Reference
Type I IFN receptor	IFNAR1	Woodchuck	IFNα/IFNβ	JN379357	[28]
		Ovis aries	IFNα/IFNβ	U65978	[27]
	IFNAR2	Bos taurus	IFNα/IFNβ	U75304	[27]
		Ovis aries	IFNα/IFNβ	U65979	[27]
		Feline	IFNα/IFNβ	JN797630	[26]
		Woodchuck	IFNα/IFNβ	JN379359	[28]
Type II IFN receptor	IFNGR1	Mouse	IFNγ	NM010511	[24]
	IFNGR2	Mouse	IFNγ	NM008338	[25]
Type III IFN receptor	IFNLR1	Mouse	IFNλ	NM174851	[3]

The present paper presents an overview of recent progress of interferons and their cognate receptor systems in birds. The gene structure and the evolutionary analysis of interferons and their cognate receptors are also discussed. In addition, this review will provide a brief summary of the interplay between interferons and interferon receptors during infection in birds.

## 2. Interferon

In birds, studies on interferons have mainly focused on chickens and ducks (Table 3). Data for other birds is scarce, and further studies are essential to uncover detailed information about avian interferons.

**Table 3:** List of the interferons identified in birds.

Taxonomy	Gene Name	Species	Accession Number	Reference
Type I IFN	IFNα	Chicken	U07868	[29]
		Duck	X84764	[30]
		Goose	AY524422	[31]
		Turkey	U28140	[32]
	IFNβ	Chicken	X92479	[33]
Type II IFN	IFNγ	Chicken	U27465	[34]
		Duck	AF087134	[35]
		Goose	AY524421	[36]
		Turkey	AJ000725	[37]
		Pigeon	DQ479967	[38]
		Pheasant	AJ001289	[37]
		Quail	AJ001678	[37]
		Guinea Fowl	AJ001263	[37]
Type III IFN	IFNλ	Chicken	EF587763	[39]
		Duck	KJ206897	[40]

### 2.1. Type I Interferon (IFN)

In mammals, type I IFNs constitute a multigene family that includes IFNα, IFNβ, IFNε, IFNκ, IFNω, IFNδ, IFNτ [41]. In the giant panda, all IFNα subtypes display antiviral activities, but each one shows different antiviral activities [20]. Comparison of the amino acid sequences of all its subtypes with huIFN-α1 and huIFN-α2 showed approximate sequence similarities of 58%–60% and 56%–59%, respectively [20]. Recombinant canine IFNε displayed potent antiviral activity on both homologous and heterologous animal cells, and shares more than 70% identity with human and porcine IFNε [23]. In the vertebrate immune system, type I interferons have pleiotropic effects on cells, which include inducing an antiviral state, inhibiting cell proliferation, modulating cell fate (survival/apoptosis), and affecting differentiation and migration [42].

Recently, significant research progress has been made on chicken type I interferon (ChIFN). It was first identified as avian interferon using an RT-PCR approach [29]. The ChIFNα was amplified from chicken liver [43], while the ChIFNβ was first identified by southern blot analysis [33] and was amplified from the cDNA of vesicular stomatitis virus (VSV)-infected DF-1 cells [44]. ChIFNα and ChIFNβ contain 20 residues that probably interact with IFNAR1 and 27 residues that interact with IFNAR2 [44]. ChIFNα and ChIFNβ show different induction potency on various sets of interferon stimulated genes (ISGs), and the stronger antiviral activity of ChIFNα is likely attributed to the higher expression levels of downstream antiviral ISGs [44]. Additionally, avian RNA tumor virus induced duck type I IFN (DuIFN) secretion in infected duck embryo fibroblasts, which drastically inhibited the multiplication of the virus [45]. DuIFN was also a potent inhibitor of duck hepatitis B virus (DHBV) [30]. The cDNA of goose interferonα (goIFNα) was amplified from Phytohaemagglutinin (PHA)-stimulated peripheral blood mononuclear cells (PBMCs) by RT-PCR [31]. Interestingly, the recombinant goose IFNα shows partial cross-species activity [31]. Furthermore, the turkey interferon (TuIFN) mRNA expression was induced by reoviral double-stranded RNA in fibroblasts [32].

**Figure 1:**
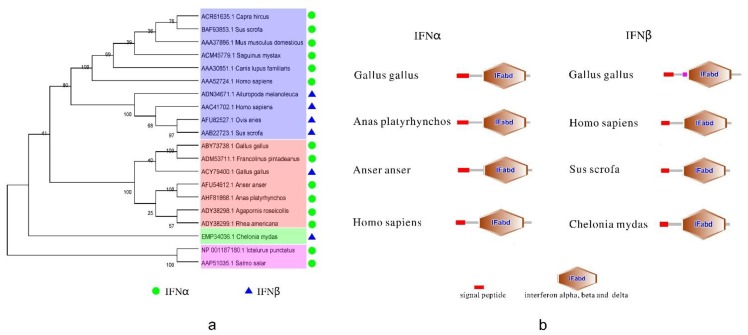
The phylogenetic tree and the protein structure of bird type I interferon (IFN). (**a**) The phylogenetic tree of type I IFN. It was built by MEGA5 program (http://www.megasoftware.net/) with 1000 bootstrap replicates and based on the neighbor-joining method. Blue, orange, green, pink in the picture represent mammals, birds, reptiles and amphibians, fish, respectively; and (**b**) The structure of bird type I IFN. IFNα reference sequences: *Gallus gallus* [GenBank: ABY73738.1], *Anas platyrhynchos* [GenBank: AHF81868.1], *Anser anser* [GenBank: AFU54612.1], *Homo sapiens* [GenBank: AAA52724.1]. IFNβ reference sequences: *Gallus gallus* [GenBank: ACY79400.1], *Homo sapiens* [GenBank: AAC41702.1], *Sus scrofa* [GenBank: AAB22723.1], *Chelonia mydas* [GenBank: EMP34036.1].

In mammals, the molecular evolution of interferon has been analyzed [46,47]. To explore the evolution of type I interferon (IFNα and IFNβ) in birds, a phylogenetic tree was built by the MEGA5 program (Figure 1a). The tree allowed us to infer that avian type I IFN and mammalian type I IFN are derived from a common ancestor, and the avian sequences are closer to mammals than to fish and reptiles. In mammals, the three-dimensional structure of the common type I interferon has 5α-helices [48,49,50]. So far, there has been little research on the structure of interferons and interferon receptors in birds. Here, the structure of bird type I IFN was predicted according to existing data using the SMART software (Figure 1b). IFNα and IFNβ from different species have some similarities in their predicted structures; they both have a signal peptide domain and a common domain of interferon α, β and δ. Based on these observations, it is suggested that the functions of interferons in birds are the same as those in mammals, reptiles, and fish; however, special structures may confer unique functions on the host. Except for IFNγ, the domains of interferon α, β and δ are related. In terms of the functions of the structural domains, the divergent features and functions of different motifs remain to be determined. However, it is anticipated that heterogeneous interferon can provide cross protection between divergent avian species. Compared to mammals, how the heterogeneous interferon might affect divergent species remains a challenge, because of the lack of novel methods and the incomplete understanding of the molecular mechanisms of the signaling pathways in birds.

### 2.2. Type II IFN

Type II interferon (IFNγ), which is a potential macrophage-activating factor [51], orchestrates maturation and differentiation of various cell types [52,53,54]. It plays an important role in the activation and regulation of innate and adaptive immunity, and is involved in the Th-1 type immune response [51,55].

Notably, chicken IFNγ (ChIFNγ) was cloned from a chicken T cell line and shared the 35% and 32% sequence similarity to its equine and human counterparts, respectively [34]. Furthermore, the early expression of IFNγ in response to infection with a densonucleosis virus (DNV) strain had a significant protective role against the effects of highly virulent Newcastle disease virus (NDV) infection in chickens [56]. Intriguingly, the expression of ChIFNγ transcripts can be significantly down-regulated by RNA interference, triggering sequence-specific gene silencing [57]. Moreover, the cDNA cloning and initial characterization of duck IFNγ homolog (DuIFNγ) has been described [35]. Importantly, treatment of primary duck hepatocytes with recombinant DuIFNγ inhibited Duck Hepatitis B virus (DHBV) replication in a dose dependent manner [35]. The goose interferon gamma (goIFNγ) cDNA was also amplified from PHA stimulated goose PBMCs by RT-PCR [36]. However, goIFNγ, compared with chicken and duck interferon, seems to be a less potent antiviral agent [36]. According to their ability to induce nitric oxide (NO), goIFNγ and goIFNα might have distinct biological functions [31,36]. Considerable evidence has accumulated revealing that even in the same species, various subtypes of interferon have distinct biological activities. The different activities of goose interferon subtypes will be determined in future studies of the identification of the genes and the immune response to viruses. By contrast, the pigeon IFNγ was cloned, and the recombinant protein was functional in chicken cells [38]. Surprisingly, pigeon and chicken IFNγ show cross reactivity [38], as do IFNγ from chicken and turkey, which share high sequence identity [58]. Coding sequences and partial intron sequences have been reported in four species: guinea fowl, ring-necked pheasant, Japanese quail and turkey [37].

**Figure 2:**
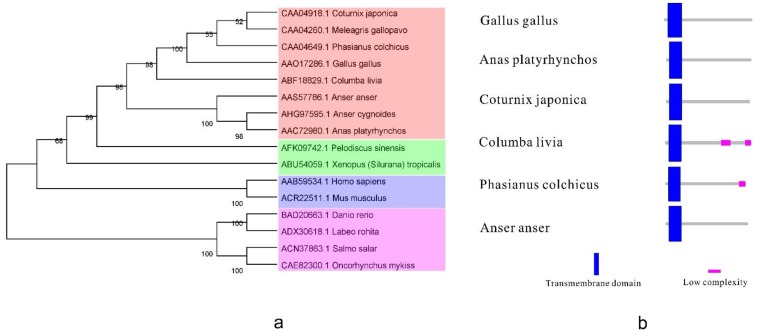
The phylogenetic tree and the protein structure of bird type II IFN. (**a**) The phylogenetic tree of type II IFN. It was built by MEGA5 program with 1000 bootstrap replicates and based on the neighbor-joining method. Blue, orange, green, pink in the picture represent mammals, birds, reptiles and amphibians, fish, respectively; and (**b**) The structure of bird type II IFN. Reference sequences: *Gallus gallus* [GenBank: AAO17286.1], *Anas platyrhynchos* [GenBank: AAC72980.1], *Coturnix japonica* [GenBank: CAA04918.1], *Columba livia* [GenBank: ABF18829.1], *Phasianus colchicus* [GenBank: CAA04649.1], *Anser anser* [GenBank: AAS57786.1].

The phylogenetic tree (Figure 2a) shows that the IFNγ from different kinds of birds are remarkably similar and that the IFNγ has been conserved in the evolution of birds. Lower vertebrate IFNγs show a striking similarity in the regions of the core structure [59]. Four exons and three introns are present in the human and chicken IFNγ genomic sequences [60]. Here, the predicted gene structure of Type II interferon shows (Figure 2b) that the transmembrane domain in birds is highly conserved.

### 2.3. Type III IFN

Similarly, Type III interferons have indispensable and unique roles, not only in antiviral immunity [61,62], but also in cancer immunotherapy [63]. The first ChIFNλ was cloned from chicken splenic leukocytes stimulated with poly-riboinosinic-ribocytidylic acid (Poly(I:C)) [39]. ChIFNλ has antiviral properties similar to those of human IFNλ [39]. ChIFNλ markedly inhibited the replication of various virus strains, including highly pathogenic influenza A viruses, in epithelium-rich tissue and in cell culture systems [64]. Furthermore, the Peking duck interferon lambda (DuIFNλ) has been identified, which revealed significant conservation of genomic organization and protein structure between avian and mammalian IFNλs [40]. However, there is little information on other avian type III IFNs in the literature. Further studies will continue to provide us with new insights into the cross-reactivity of avian type III IFNs and whether they perform the crosstalk between related signaling pathways.

**Figure 3:**
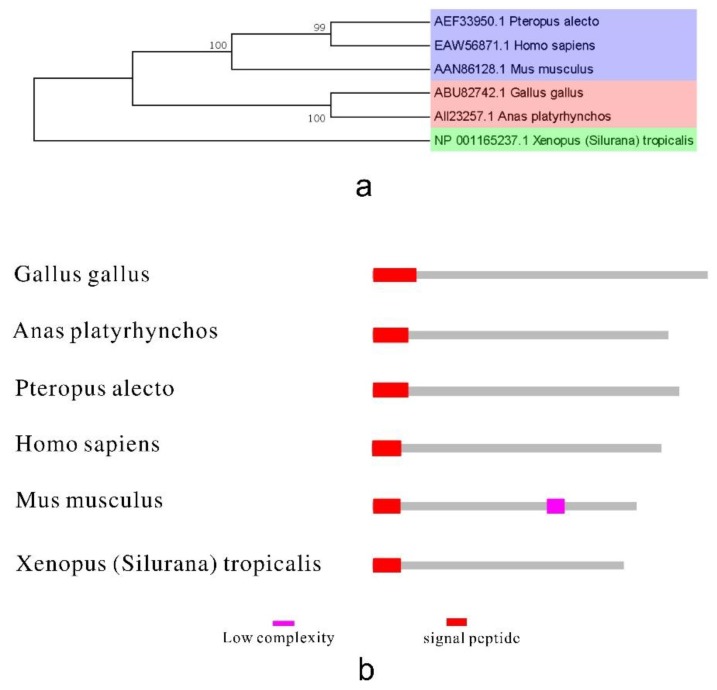
The phylogenetic tree and the protein structure of bird type III IFN. (**a**) The phylogenetic tree of type III IFN. It was built by MEGA5 program with 1000 bootstrap replicates and based on the neighbor-joining method. Blue, orange, green in the picture represent mammals, birds, reptiles and amphibians, respectively; and (**b**) The structure of bird type III IFN. Reference sequences: *Gallus gallus* [GenBank: ABU82742.1], *Anas platyrhynchos* [GenBank: AII23257.1], *Pteropus alecto* [GenBank: AEF33950.1], *Homo sapiens* [GenBank: EAW56871.1], *Mus musculus* [GenBank: AAN86128.1], *Xenopus (Silurana) tropicalis* [GenBank: NP_001165237.1].

Research on type III IFN in birds lags behind that of the other interferons. Type III IFNs from birds differ from mammals and reptiles, based on the phylogenetic tree (Figure 3a). Both mammalian and ChIFNλ genomic structures are organized into five exonic regions [39]. Here, the gene structure was predicted according to current data using the SMART software (http://smart.embl-heidelberg.de/) (Figure 3b). The predicted structures of IFNλ in divergent animals are almost the same in similar positions. Although interferons in birds have not been characterized comprehensively, a limited number of sequences of avian IFNλ are available in databases. However, the cDNA sequences and gene characterization of birds IFNλ remain to be clarified, as does the molecular mechanisms of its antiviral activity.

### 2.4. Ontogeny of Avian Interferons

The expressions of interferon-related genes were more pronounced in the larger Japanese flounder (*Paralichthys olivaceus*) [65]. Upon virus infection, the basal expression levels of virus recognition proteins were higher in adult fish [65]. In zebrafish, the embryos quickly died post-infection; however, the overexpression of IFN increased their survival rate [66]. Embryonic susceptibility to viruses may reflect a deficient interferon system. Furthermore, the ontogeny and development of innate immunity in swine has been discussed [67]. The immune system of neonates is functionally different from that of adults because of the immature system of T cells or B cells pool [67]. The innate immune response is affected by human aging, as a consequence of the changes in the expressions of innate immune cell receptors [68]. In addition, an effective and robust immune response in adult animals is likely caused by the mature defensive system. By contrast, neonatal and young animals may be more susceptible to viral infection. Newly hatched chickens appear to be more susceptible to infections than mature chickens [69]. In birds, the juvenile period is very important for immune development; however, it may be weaker in this phase than in other stages. Previous studies revealed that adult avians are more resistant to virus invasion and interference; however, whether the expression level of interferons or interferon receptors determine this capacity is unknown. It is important to explore whether the ontogeny of interferon and its cognate receptors are consistent during development and viral infection. This may contribute to a better understanding why the neonatal birds were more susceptible to pathogens than mature birds. It is predicted that the dominating factor may be the interferon receptors, because they may govern positive feedback to enlarge the immune state.

The tissue distribution profiles of interferons may be related to the age of the animals [69,70]. The expression of IFNγ was higher in the spleen of post-hatch chickens compared with that in chick embryos [70]. Additionally, DuckIFNα was induced in all organs following DHV-1 infection and varied according to the age of the ducks [71]. It is predicted that the universal distribution of IFN in immune tissues is associated with its immune defense function. The expression of this kind of immune cytokine might be highly dynamic in various types of tissues.

## 3. Interferon Receptors

The interferon receptors belong to the family of Class II helical cytokine receptors [72], which were mainly identified in humans and mice. In mammals, the biochemical and biological functions of type I interferon receptors are known [73]. In addition, the type II and type III interferon receptors are involved in antiviral immune pathways. Interferon receptors knockout models have made significant contributions to elucidating their associated immune mechanisms [74,75]. Recently, studies of interferon receptors have contributed vital clues for the comprehensive understanding the interferon signaling pathways and the explanation of their protein-protein interactions.

In birds, only the chicken interferon receptor has been reported (Table 4); however, some predicted sequences of interferon receptors from birds are present in NCBI, such as *Pseudopodoces humilis* (XP_005526691.1), *Geospiza fortis* (XP_005427851.1), *Falco cherrug* (XP_005438665.1), *Falco peregrinus* (XP_005234444.1),* Melopsittacus undulatus* (XP_005151847.1) and *Columba livia* (XP_005511566.1). Interferon receptors in birds have not yet been characterized at the molecular level, so there is still a long way to go toward a complete understanding of the signal recognition mechanism that involves the interferon receptor and the subsequent antiviral response of birds, especially in waterfowl. The various type of interferon receptor may be regulated in various and connected ways. To decipher the mechanisms of receptor regulated transcription in birds, further molecular and functional identifications of interferon receptors are required.

**Table 4:** List of the interferon receptors identified in birds.

Taxonomy	Gene Name	Species	Ligand	Accession Number	Reference
Type I IFN receptor	IFNAR1	Chicken	IFNα/IFNβ	AF082664	[76]
	IFNAR2	Chicken	IFNα/IFNβ	AF082665	[76]
Type II IFN receptor	IFNGR1	Chicken	IFNγ	EU057149	[77]
	IFNGR2	Chicken	IFNγ	AY957508	[78]
Type III IFN receptor	IFNLR1	Chicken	IFNλ	419694(Gene ID)	[79]
	IL10R2	Chicken	IFNλ	AF082666	[76]

### 3.1. Type I IFN Receptors

**Figure 4:**
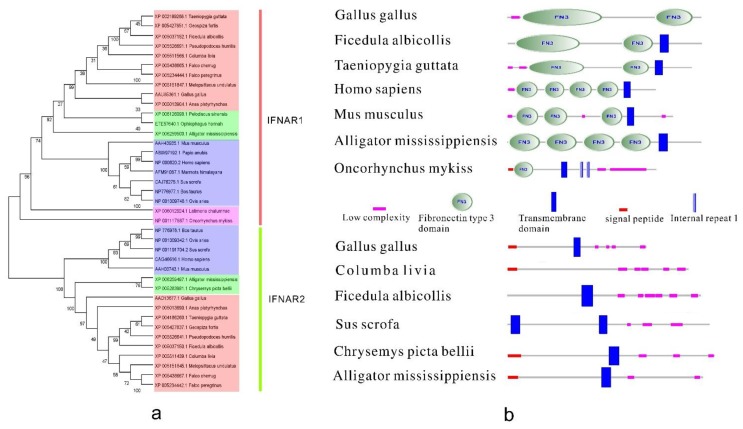
The phylogenetic tree and the protein structure of bird type I IFN receptors. (**a**) The phylogenetic tree of type I IFN receptors. It was built by MEGA5 program with 1000 bootstrap replicates and based on the neighbor-joining method. Blue, orange, green, pink in the picture represent mammals, birds, reptiles and amphibians, fish, respectively; and (**b**) The structure of bird type I IFN receptors. IFNAR1 reference sequences: *Gallus gallus* [GenBank: AAU85361.1], *Ficedula albicollis* [GenBank: XP005037152.1], *Taeniopygia guttata* [GenBank: XP002189268.1], *Homo sapiens* [GenBank: NP000620.2], *Mus musculus* [GenBank: AAH43935.1], *Alligator mississippiensis* [GenBank: XP006259500.1], *Oncorhynchus mykiss* [GenBank: NP001117887.1]. IFNAR2 reference sequences: *Gallus gallus* [GenBank: AAD13677.1], *Columba livia* [GenBank: XP005511439.1], *Ficedula albicollis* [GenBank: XP005037150.1], *Sus scrofa* [GenBank: NP001191704.2], *Chrysemys picta bellii* [GenBank: XP005283981.1], *Alligator mississippiensis* [GenBank: XP006259497.1].

Chicken IFNAR1, IFNAR2 and IL10R2 were identified by comparative genomic analysis [76]. However, the unique functions of each individual subunit of the interferon receptors in birds remain to be elucidated. Despite considerable progress in the molecular cloning of type I IFN receptors in chickens, numerous questions regarding the other receptors remain unanswered. The phylogenetic tree (Figure 4a) shows that the type I IFN receptor gene is conserved in birds and in many higher vertebrates, especially among animals that are closely related. Although interferon receptors play a critical part in signaling, relatively little is known about their structural domains. The structures of the type I IFN receptor in birds and mammals are shown in Figure 4b. The high consistency of their composition and similarities in their major constituents indicate a close correlation between type I IFN receptors from birds and other species. IFNAR1 and IFNAR2 form a distinct clade; however, this is phylogenetically close to a large family. The two subunits of the interferon receptor form a distinct cluster; thus, the orthologs (IFNAR1 and IFNAR2) may be derived from the same ancestral gene. This may be attributable to the presence of multiple copies of the primitive interferon receptor gene.

### 3.2. Type II IFN Receptors

**Figure 5:**
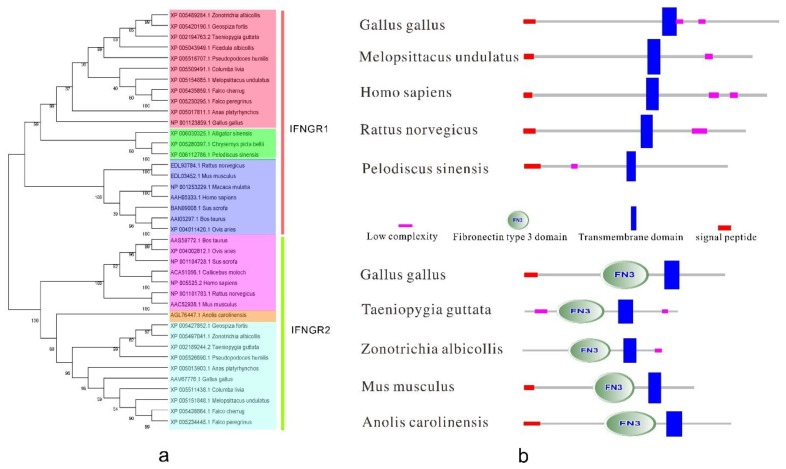
The phylogenetic tree and the protein structure of bird type II IFN receptors. (**a**) The phylogenetic tree of the type II IFN receptors. It was built by MEGA5 program with 1000 bootstrap replicates and based on the neighbor-joining method. For IFNGR1, dark orange, green, pink in the picture represent birds, reptiles and amphibians, mammals, respectively. For IFNGR2, pink, light orange, light blue in the picture represent mammals, reptiles and amphibians, birds, respectively; and (**b**) The structure of bird type II IFN receptors. IFNGR1 reference sequences: *Gallus gallus* [GenBank: NP001123859.1], *Melopsittacus undulatus* [GenBank: XP005154885.1], *Homo sapiens* [GenBank: AAH05333.1], *Rattus norvegicus* [GenBank :EDL93784.1], *Pelodiscus sinensis* [GenBank: XP006112786.1]. IFNGR2 reference sequences: *Gallus gallus* [GenBank: AAV67776.1], *Taeniopygia guttata* [GenBank: XP002189244.2], *Zonotrichia albicollis* [GenBank: XP005497041.1], *Mus musculus* [GenBank: AAC52938.1], *Anolis carolinensis* [GenBank: AGL76447.1].

cDNA sequences of chicken interferon-γ (IFNγ) receptor α-chain (ChIFNGR1) and β-chain (ChIFNGR2) were cloned using rapid application of cDNA ends (RACE) [77,78]. The phylogenetic tree (Figure 5a) shows that IFNGR1 and IFNGR2 may be derived from the same ancestral gene. The structure was predicted according to selected data form different species using the SMART software (Figure 5b). Surprisingly, IFNGR2 has a fibronectin type III domain (FN3), while IFNGR1 does not. Fibronectins are multi-domain glycoproteins found in a soluble form in the plasma, and in an insoluble form in loose connective tissue and basement membranes [80]. Perhaps this approximately 100 amino acid domain of IFNGR2 provides a special function that complements that of IFNGR1.

### 3.3. Type III IFN Receptors

**Figure 6:**
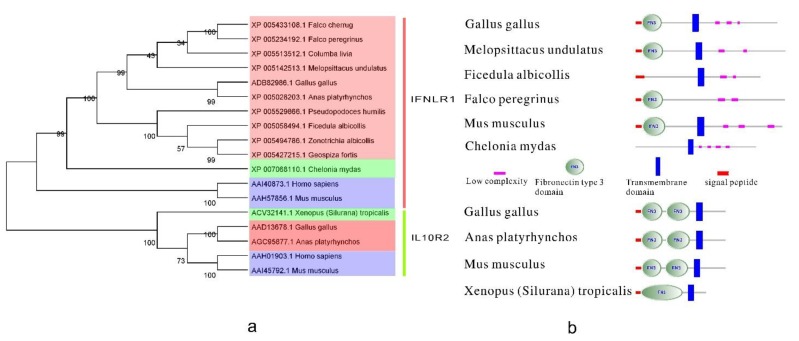
The phylogenetic tree and the protein structure of bird type III IFN receptors. (**a**) The phylogenetic tree of the type III IFN receptors. It was built by MEGA5 program with 1000 bootstrap replicates and based on the neighbor-joining method. Orange (dark or light), green, blue in the picture represent birds, reptiles and amphibians, mammals, respectively; and (**b**) The structure of bird type III IFN receptors. IFNLR1 reference sequences: *Gallus gallus* [GenBank: ADB82986.1], *Melopsittacus undulatus* [GenBank: XP005142513.1], *Ficedula albicollis* [GenBank: XP005058494.1], *Falco peregrinus* [GenBank: XP005234192.1], *Mus musculus* [GenBank: AAH57856.1], *Chelonia mydas* [GenBank: XP007068110.1]. IL10R2 reference sequences: *Gallus gallus* [GenBank: AAD13678.1], *Anas platyrhynchos* [GenBank: AGC95877.1], *Mus musculus* [GenBank: AAI45792.1], *Xenopus (Silurana) tropicalis* [GenBank: ACV32141.1].

There is little information about type III IFN receptors. A predicted sequence for the IFNLR1 subunit was derived by automated computational analysis using gene prediction program GNOMON [79]. The phylogenetic tree (Figure 6a) indicates that type III IFN receptors from birds have high homology. The diagram of their structure (Figure 6b) showed that all identified type III IFN receptors contain a signal peptide, a transmembrane domain, and an FN3 domain (except for those from *Ficedula albicollis* and *Chelonia mydas*), which indicates low complexity. The structures of IFNLR1 and IL-10R are conserved to some extent.

### 3.4. Ontogeny of Avian Interferon Receptors

The ontogeny of type I and III receptor subunits expression has been discussed [81]. It is predicted that the ontogeny of interferon receptors is related to age. With the development of interferon receptors and increasing age, the immune mechanism of the host matures. Interferon receptors play important roles in defense against viruses or other pathogens utilizing the positive feedback effect of IFNs through interferon receptors. This may explain why adult animals are more resistant to virus invasion and pathogenic interference.

## 4. Antiviral Molecular Mechanism of Interferon and Interferon Receptor Activity

Different kinds of pattern recognition receptors (PRRs) sense different viral pathogen-associated molecular patterns (PAMPs), thus producing mass interferon release against infection. Extraordinary advances have been made in the last decade in our knowledge of pattern recognition receptors and cytosolic receptors in birds [82]. The level of interferon production is based on the function of these initial PAMPs in the first wave. Agonists that work as antiviral drugs may have potential antiviral activity by stimulating the signaling pathway to produce interferon.

### 4.1. Interferon Functions as the Master Key

Interferon can be regarded as the master key, which could turn in the lock to achieve strong and significant antiviral immunity [42,83]. Interferons, especially type I interferons, have been described as the master factors that bridge innate and adaptive immunity [42,83,84]. In vertebrates, IFN is released after the activation of PRRs; interferon then binds to the interferon receptor, leading to a series of products, including some immune proteins and other subtypes of interferons. The first wave of interferon expression is triggered by viral infection, and the second wave may be produced to boost the host defense system via the IFN-IFN receptor interaction. Thus, interferons resemble a key that can control the antiviral immune response of the host. Currently, research attention has mainly concentrated on the interaction between viruses and PRRs [82]. However, the recognition and the interaction between interferons and their associated interferon receptors in birds remain unknown.

ChIFNα and ChIFNβ, which are induced by several viruses, such as Sendai virus (SeV) [44], vesicular stomatitis virus (VSV) [44] and influenza virus (H5N1) [81], show differential virus-induced mRNA production. Early production of high levels of IFNγ, induced by virulent NDV replication, is able to attenuate the pathogenicity and tissue damage [56]. The production of type III IFN exhibits a similar mechanism to type I IFN in mammals [61]; however, the mechanism of activation in birds is unknown. Infection with Duck hepatitis virus (DHV) induced the upregulation of IFNα gene expression in ducks [71]. Additionally, goIFNα and goIFNγ were induced in PHA-stimulated peripheral blood mononuclear cells (PBMCs) [31,36]. It may be inferred that the different kinds of viruses or stimulants can induce the production of diverse types of interferons in birds. Additionally, IFNγ binds to IFNGR1 and IFNGR2 to induce a downstream antiviral signal. This subtype of interferon in birds may act as another key, which can turn the receptors on or off. To some extent, type III IFN could also be regarded as a similar key. Probably because of the relatively limited information about the mechanism in birds, there is little information on whether and how the key activates the receptor. Different subtypes of interferons may take part in disparate pathways and exhibit individual antiviral effects. Importantly, they may both make a combined contribution to the immune system in birds.

**Figure 7:**
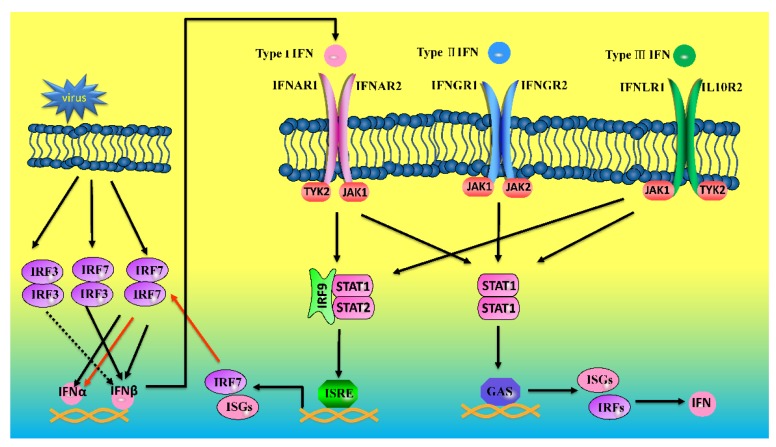
Sketch map of the possible positive feedback antiviral signal pathway of IFN-IFNR in birds.

Type I IFNs bind to the heterodimeric receptor (IFNAR1 and IFNAR2) to induce subsequent activation of proteins. In many cells, type I IFNs activate a major transcription factor, ISGF3, a complex of phosphorylated STAT1, STAT2, and interferon regulatory factor 9 (IRF9) (Figure 7), which binds to IFN-stimulated response elements (ISREs) present in the promoters of many ISGs [85]. Type I and type III interferons have a similar pattern in the signaling pathway of interferon receptor binding to achieve the antiviral state (Figure 7). The mammalian JAK family consists of four members: JAK1, JAK2, JAK3 and tyrosine kinase 2 (TYK2) [86], which are associated with interferon receptors. Once phosphorylated, signal transduction and activators of transcription (STATs) will be phosphorylated when recruited to the docking sites of receptors, which are then dimerized and translocated to the nucleus to activate transcription of ISGs [87]. Finally, some ISGs and inflammatory cytokines were highly expressed to initiate a host immune response.

### 4.2. Interferon Receptors Function as the Signal Locks

In the induction of antiviral immunity, the core molecules are the interferon receptors. It is likely that the three types of receptors may differ significantly in their signal transduction. However, the mechanism of recognizing the upstream and downstream cytokines may have a lot in common.

Interferon receptors, just like a signal molecular switch, play an important role in regulating the immune response against foreign pathogens. Furthermore, studies using primary murine cells that lacked IFNAR1 showed that IFNAR1 is required for complete binding and signal transduction of interferon [88]. Mouse embryonic fibroblasts lacking the type I interferon receptor exhibited decreased levels of transcription of antiviral genes [89]. Once this signal lock opens, the defensive line of the host will be strengthened. Furthermore, the expression level of an interferon receptor is closely related to numerous types of factors. The consequent effect of the suppressor of cytokine signaling 1 (SOCS1) negatively affects IFNAR1 surface expression though interaction with Tyk2 [90]. It worth mentioning that IL-29 raised, whereas IFNα reduced, the expression of IFNGR1 on the surface of macrophages [91]. This showed that IFNGR1 is affected by type I interferon. To date, there is little information on whether and how IFNLR1 is regulated after the IFNλ signal cascade. As a subunit of IFNLR, IL10R2 may have a role in the regulation of IFNLR1 expression. Whether IFNRs are affected and regulated by other cellular proteins has yet to be addressed. It may be that the positive feedback of interferon decreases if the receptor cannot work properly. However, in birds, a series of studies on the detailed system of IFNR regulation are required.

The activation of an interferon receptor leads to the release of IRFs. In vertebrates, IRF3 and IRF7 play prominent roles in the regulation of second wave production of interferon [14]. However, in birds, IRF3 is thought to be missing [92,93]. To date, there has been little research concerning IRFs in birds, and how these factors regulate the interferon remains elusive.

After the release of IRFs, ISGs are induced. In mammals, many ISGs have important roles in the defense against viral infection, such as hepatitis B virus [94], vesicular stomatitis virus [95] and West Nile Virus [96]. Mx proteins limit viral gene expression partly though blocking its transcription [97] and by binding to viral nucleocapsids to block the production of progeny virus [98]. Protein kinase-R (PKR) is the only member of the eukaryotic translation initiation factor 2-α (eIF2-α) kinase family that is induced by interferon; it exerts antiviral activity by preventing viral protein synthesis [99]. Interferon-inducible transmembrane protein 3 (IFITM3) can restrict replication of viruses by affecting viral entry steps, such as binding and fusion with cell endosome membranes [100]. However, in chickens, Mx and PKR failed to protect chickens from highly pathogenic avian H5N1 influenza virus infection [101]. By contrast, chicken IFITM3 restricts cell infection by influenza A viruses and lyssaviruses [102]. In addition, 2'-5' oligoadenylate synthetase and Mx1, induced by ChIFNα, actively participate in regulating the anti-AIV response [43]. However, there may be more, as yet undiscovered, ISGs in birds that exhibit divergent antiviral function; thus, there is a long way to go toward a complete understanding of ISGs signal pathway in birds.

Although there have been considerable advances in molecular cloning and the characterization of different types and subtypes of IFNs, complex and volatile interferon receptor-mediated interferon signaling pathways and predicted alternative pathways is a novel field of research in birds. Thus, greater knowledge of the events that result in regulation of interferon receptors requires further research.

### 4.3. The Positive Feedback Antiviral Effect

In mammals, upon viral infection, the secretion of interferon occurs in the early phase, leading to IRF7 activation in the late phase by stimulating the JAK-STAT pathway through the interferon receptor system [14] (Figure 7). Then, hundreds of IFN-stimulated genes are induced and antiviral immunity is enhanced [41,84]. Note that IRF7 is the master regulator of type I IFN gene induction [103]. Nevertheless, there are few reports of this special molecular mechanism in birds, however, it can be inferred that a similar mechanism may be present in birds. Recombinant ChIFNα treatment significantly upregulated endogenous ChIFNα and ChIFNβ, while the positive feedback effect of ChIFNβ was much lower [44]. Thus, interferons in birds may have a self-control system to induce interferon. Whether IRF7 or other IRFs are involved remains unknown. Importantly, exploitation of the positive effect of interferon might help ameliorate certain infectious disease in the poultry industry.

Interestingly, in mice, the type I IFN system provides feedback not only on its own expression, but also on type III IFN expression [104]. However, whether the birds exhibit this crosstalk loop during infection remains unknown. Additionally, whether the ability to make a timely and effective immune response is derived from the molecular mechanism of positive feedback is unknown. Thus, the interferon feedback pathway of birds should be studied in further detail. Utilizing their positive feedback antiviral effect, related receptors are likely candidates for novel therapeutic agents.

### 4.4. The Dynamic Balance of Immune Regulation in Vivo

**Figure 8:**
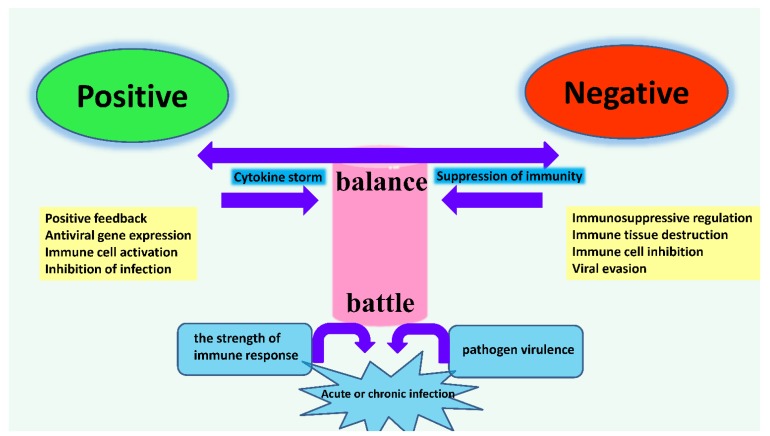
The sketch map of dynamic balance of immune regulation in birds. Interferon maintains a balance in antiviral actions against immunosuppressive effects during viral infection. The precise control of interferon balance is critical for efficient viral clearance without harmful immunopathology. Both sides of interferon regulation (positive and negative) can be affected mutually by the strength of immune response and pathogen, like an immune battle. In immune regulation of host, both the cytokine storm and suppression of immunity should be avoided.

The study of the regulation of interferon and interferon receptor signaling is an important research area, because unbalanced signaling may contribute to immune diseases. Notably, this equilibrium is not only maintained in pathological, but also under physiological circumstances (Figure 8). To diminish deleterious autoimmune responses those involved in the regulation of IFN receptor levels on the surface of target cells have been elucidated [4]. In birds, is this process passive or facilitated? Of particular interest is the balance during infection between infection and immunity. In the interferons-related immune system, both the cytokine storm and suppression of immunity should be avoided. This immune battle should be balanced by both the strength of the immune response and the pathogen’s virulence (Figure 8). Taken together, disequilibrium in birds may provide the chance for foreign pathogens, such as bacteria or viruses, to undermine the state of the hosts.

Balanced responses in the host are appropriate for eradication of pathogens and to alleviate autoimmune diseases [83]. Interference of IFNγ receptor complex gene expression in MDV-infected chickens revealed that MDV evades host attack, which may prevent the activation of the antiviral pathway by directly reducing IFNγ receptor expression [105], thus influencing the balance of interferon and interferon receptor. In bats, a study showed that suppression of type I IFN was accompanied by the induction of type III IFN after virus infection [106]. Thus, type I interferon elicits seemingly opposite, yet interrelated, positive and negative influences on virus replication and dissemination [107]. Hepatitis B virus (HBV) interferes with IFNAR signal transduction, partly by decreasing the level of IFN receptors, thus attenuating interferon triggered antiviral signal transduction [108]. Additionally, the inhibition of immune related cytokines is one of the reasons behind the pathogenesis observed upon infection by virulent strains. Conversely, attenuated strains may induce high levels of these cytokines, which provides a solid theoretical basis for exploiting novel vaccines [12]. One possible explanation for these opposite effects might be interferons’ functions in defense foreign attack and that the mechanisms of viruses that control these functions are continuously evolving.

Birds, including chickens, ducks and geese, are natural reservoirs for many kinds of viruses. It can be speculated that viruses can coexist with interferons in birds; meanwhile, different types of interferons or interferon receptors may show divergent trends. It is vital to obtain further knowledge of avian interferons and interferon receptors, which will promote effective strategies and novel vaccines for the prophylaxis and therapy of infectious diseases.

## 5. Conclusions

The complex structures of interferons and their receptors, as well as the mechanisms of protein–protein interactions between interferons and their receptors should be studied further. The interferon and interferon receptor systems in birds have a lot in common with other vertebrates, such as their genetic relationships in evolution and gene structure. In terms of its subtle structure, interferon receptors are more complicated than interferons, and the signal lock pathway of interferon receptor subunits should be emphasized.

The auto-amplification loop is the core of this complex signaling pathway in the defense against foreign pathogens. Notably, crosstalk between interferons and their receptors in birds has not yet been reported. Such feedback may be used to combat avian viruses and decrease the transmission of zoonotic viruses, especially in poultry. Further studies are also needed to fully elucidate the various mechanisms by which the interferon response coexists with the virus. Furthermore, interplay between interferons and interferon receptors under physiological or pathological states may be a balanced system. Immune system disorders can lead to autoimmune diseases and damage after viral attack; therefore, keeping a balanced system is required for an effective immune response.

In summary, the coordination and cooperation of multiple distinct signaling cascades in birds, mediated by different kinds of interferons, as well as interferon receptors, remains a riddle and the interplay between them remains to be determined.
